# Viral Subversion of the Chromosome Region Maintenance 1 Export Pathway and Its Consequences for the Cell Host

**DOI:** 10.3390/v15112218

**Published:** 2023-11-06

**Authors:** Makram Mghezzi-Habellah, Léa Prochasson, Pierre Jalinot, Vincent Mocquet

**Affiliations:** Laboratoire de Biologie et Modélisation de la Cellule, Ecole Normale Supérieure-Lyon, Université Claude Bernard Lyon, U1293, UMR5239, 69364 Lyon, France; makram.mghezzi-habellah@ens-lyon.fr (M.M.-H.); lea.prochasson@gmail.com (L.P.); pierre.jalinot@ens-lyon.fr (P.J.)

**Keywords:** virus, exportin CRM1/XPO-1, nuclear export, nucleocytoplasmic trafficking, viral infection, viral hijacking

## Abstract

In eukaryotic cells, the spatial distribution between cytoplasm and nucleus is essential for cell homeostasis. This dynamic distribution is selectively regulated by the nuclear pore complex (NPC), which allows the passive or energy-dependent transport of proteins between these two compartments. Viruses possess many strategies to hijack nucleocytoplasmic shuttling for the benefit of their viral replication. Here, we review how viruses interfere with the karyopherin CRM1 that controls the nuclear export of protein cargoes. We analyze the fact that the viral hijacking of CRM1 provokes are-localization of numerous cellular factors in a suitable place for specific steps of viral replication. While CRM1 emerges as a critical partner for viruses, it also takes part in antiviral and inflammatory response regulation. This review also addresses how CRM1 hijacking affects it and the benefits of CRM1 inhibitors as antiviral treatments.

## 1. Introduction

While small molecules (<40 kDa) can diffuse passively through the nuclear pore complex (NPC), the transport of macromolecules called “cargoes” mainly relies on the nuclear transport receptors of the Karyopherin β family (Kaps). Kaps include nuclear import receptors called importins, nuclear export receptors called exportins, and bidirectional receptors. Their role is to cross the NPC thanks to concomitant interactions with the cargoes and the nucleoporins (Nups) of the NPC [1]. Concerning the export, CRM1/XPO-1 (chromosomal region maintenance 1/exportin 1) has the largest interactome out of the seven exportins described before. CRM1 was first identified in *Schizosaccharomyces pombe* by the characterization of a mutation leading to a phenotype of distorted chromosomes, earning the name of Chromosome Region Maintenance 1 [2]. CRM1 was then characterized as a nuclear receptor, thanks to the discovery of a natural and selective inhibitor of its export, named Leptomycin B (LMB) [3,4], and the identification of the viral protein Rev from HIV-1 as an adapter protein for the export of viral RNAs [5,6,7]. The role of CRM1 in the export of specific cellular RNAs pre-associated with protein cargoes emerged later. Since then, the use of CRM1 synthetic inhibitors has highlighted the need for strict control of the cellular proteins nucleocytoplasmic distribution to ensure cellular homeostasis and healthy behavior. As a consequence, a great diversity of biological functions controlled by this exportin was further identified.

In the context of infection, viral proteins invade most of the cellular compartments in order to complete viral replication and suppress the host antiviral threats. Consequently, they actively shuttle between nucleus and cytoplasm; thus, hijacking the CRM1-dependent export emerges as a common phenomenon to divert cell functioning in favor of viruses, although the molecular mechanisms underlying this are virus-specific. Our knowledge of the different cellular processes involved in this hijacking is still lacking. Here, we exhaustively review the current literature on these matters: we focus on the mechanisms of inhibition or hijacking favoring viral replication as well as the possible impacts of these process for the host. First, we present the CRM1-dependent export mechanism and its related cellular functions. Then, we describe how interactions between viral proteins and CRM1 promote the export of vRNA, induce the transport of viral components, modify the nuclear pore composition, or directly relocate nuclear host proteins for the benefit viral replication. Finally, we explore the biological process that may be altered from this CRM1 hijacking. 

## 2. CRM1-Dependent Export of Macromolecules

### 2.1. Association Cargo—CRM1: The NES Pattern

**Figure 1:**
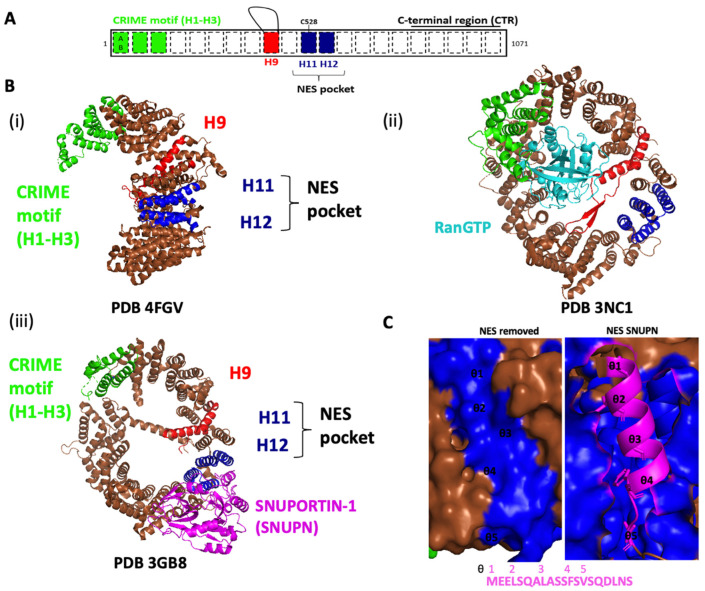
Structure of CRM1. (**A**) Schematic representation on CRM1 showing its important motifs. In N-ter, the CRIME motif is colored green. The regulatory heat 9 (H9) is red. The NES pocket (H11 and H12) is blue. Colors are respected in all the different structures. (**B**) Different experimental crystal structure of CRM1. (i) “Open” structure of CRM1 with low affinity for the cargoes. RCSB PDB: 4FGV from [16]. (ii) Ring structure of CRM1 in association with RanGTP (in cyan), required for active export to cytoplasm. RCSB PDB: 3NC1 from [17]. (iii) Structure of the export complex with high affinity for the cargo, Snuportin (SNUPN—magenta). SNUPN interacts with the NES pocket but additional contacts with CRM1 backbone are required for the stabilization of the complex. To facilitate the visualization, RanGTP has been removed. RCSB PDB: 3GB8 from [18]. (**C**) Magnification of the SNUPN NES (magenta) orientation in the NES-binding groove of CRM1. Hydrophobic acids (Φ) from the NES sequence are indicated. All the structures were generated using PyMOL Molecular Graphics System Version 2.5.5.

In order to interact with a specific cargo and address it to the cytoplasm, CRM1 recognizes a particular motif called NES (nuclear export signal) [8,9]. Numerous structural studies determine a NES consensus sequence as follows: Φ0-(x)2-Φ1-(x)1-2-3-Φ2-(x)2-3-Φ3-x1-Φ4. Here, Φ represents hydrophobic residues and x represents any amino acid. Based on these hydrophobic residues and their spacing, NES adopt specific conformations called “binding groove” that define “NES classes” [1,10,11,12]. While the experimentally calculated affinities (KD) vary greatly between the different classes (KD _Rev-NES_ of class 2 = 1180 nM; KD _SNUPN-NES_ of class 1c = 12,500 nM), they also vary within the same class (KD _PKI-NES_ of class 1a = 34 nM; KD _STRAD-NES_ class 1a = 10,300 nM) [13], suggesting that the interaction surface between CRM1 and its cargo is broader than just the NES and modulates the overall affinity, as observed with the RanGTP–CRM1–Snurportin complex (Figure 1) [14]. Regardless, the affinity for CRM1 for most NES domains is rather low, consistent with the need to deliver the cargo protein efficiently into the cytoplasm [12,14,15].

### 2.2. Association Cargo—CRM1: The NES Pattern

To load cargoes on the nuclear side, CRM1 needs the small G protein, RAN, bound to GTP. A nucleocytoplasmic gradient of RanGTP/RanGDP is maintained with RCC1 (regulator of chromosome condensation 1) converting RanGDP to RanGTP in the nucleus, whereas RanGAP (GTPase-activating protein) dephosphorylates RanGTP into RanGDP at the cytoplasmic face of the NPC [19,20]. While independent interactions of CRM1 with either RanGTP or with its cargo are weak, the connection of CRM1 with both partners together stabilizes the formation of an export complex (cargo–CRM1–RanGTP) [1,19,21]. Several structural characteristics of CRM1 are important for the establishment of these cooperative interactions (Figure 1): CRM1 is composed of 21 domains of HEAT repeats (H1 to H21), motifs which are structured in tandem “helix A–loop–helix B” motifs—the two antiparallel alpha helices A and B of each HEAT repeat stack and align in such a way that the A type helixes form a convex outer surface and the B types helixes form a concave inner surface. H11 and H12 form a hydrophobic pocket on the outer surface of CRM1, called “NES pocket” that specifically interacts with NES motifs. In the “NES pocket”, the cysteine 528 was identified to covalently bind with Leptomycin B (LMB), leading to the selective and non-competitive inhibition of CRM1 export [22]. In addition, the C-terminal region (CTR) and the H9 loop exert an inhibitory effect by masking the H11 and H12 domains. At the N-terminal of the protein, the H1–H3 motifs form a CRIME domain involved in RanGTP binding. RanGTP binding to CRM1 induces conformational changes: the N-terminal and C-terminal extremities are brought closer, folding CRM1 in a ring-like shape, releasing the H9 loop and CTR inhibition [1,19,23,24]. Recently, the STK38 kinase was proposed to stabilize this active conformation through the phosphorylation of the CTR S1055 [25].

By interacting with these structural elements, other factors will be able to modulate the stability of the export complex. For example, it is shown that RanBP3 also binds the H9 loop and stabilizes the export complex, in particular by increasing the binding affinity of RanGTP to CRM1 in the nucleus [26]. The nuclear pore (NPC) is an assembly of ~1000 proteins comprising multiple copies of 34 different Nups. Interactions between specific Nups and the export complex drive this latter to the cytoplasm. For instance, CRM1 was shown to interact with Nup153, p62, Nup98, Nup214, and hCG1 [27]. In the cytoplasm, Ran interacts with Nup358/RanBP2 and RanBP1. In addition, the SUMOylation of RanGAP favors its recruitment to RanBP2 and stabilizes the link with RanGTP [28,29]. The reversion of RanGTP in RanGDP destabilizes the CRM1–Ran interaction, leading to the return of CRM1 into its original autoinhibitory conformation. As a consequence, the cargo dissociates from CRM1 and is released in the cytoplasm, while the exportin shuttles back into the nucleus.

## 3. Biological Functions of CRM1

**Figure 2:**
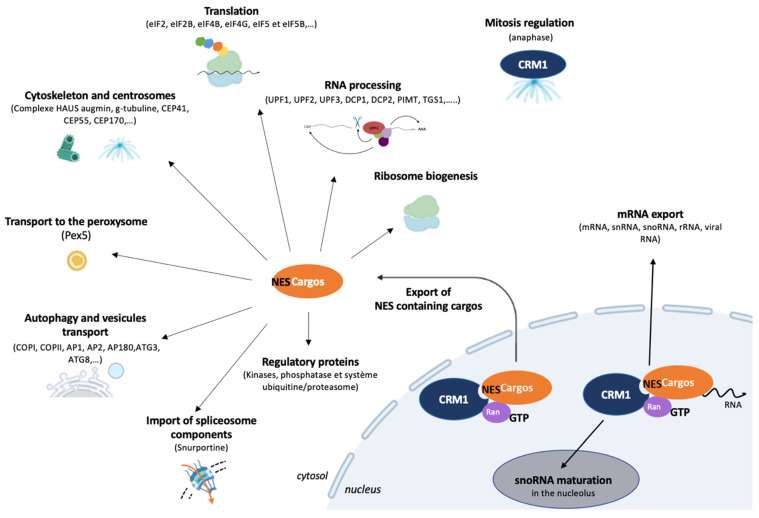
Biological process relying on CRM1. CRM1 exports protein cargoes containing NES. The multiplicity of the biological process dependent on CRM1 were revealed by the study of CRM1 mutants or overexpression in diseases, CRM1 inhibition by synthetic inhibitors and CRM1 interactome. Regulatory proteins include transcription factors that indirectly link CRM1 with several additional cellular functions. These transcriptional effects can also be combined with the RNA export function of CRM1. Overall, CRM1 regulates biological processes through time and space, ensuring the correct localization of proteins for their respective function, but also guarantees the limited residence of active factors in the nucleus to safeguard cellular homeostasis. CRM1 also exerts export-independent functions during snoRNA maturation to target snoRNA to the nucleolus.

Over the years, deciphering the biological functions and process regulated by CRM1 has been performed using three different approaches (Figure 2).

### 3.1. Evaluation of the Impact of CRM1 Associated Mutations or Overexpression in Disease

Several CRM1/XPO1 mutations were found in solid and hematopoietic tumors. A large-scale study, which uses whole-exome genomic sequencing data from 42,793 patients, identified three frequently mutated CRM1 amino acids: E571, R749, and D624 [30,31,32]. Although their functional implications in the associated oncogenesis processes are poorly understood, their analysis highlighted some of CRM1 functions. Notably, two recent studies focused on characterizing the mutant CRM1^E571K^ found in type B lymphoblastic cells. Miloudi et al. show that CRM1^E571K^ accumulates in the cytoplasm and find an abnormal association with certain partners, in particular with importin β1 [33]. Baumhardt et al. combined computing analysis and cellular localization to suggest that EIF4E-transporter (4E-T)’s affinity with CRM1^E571K^ was decreased by a factor of 10 compared to the affinity with wild-type CRM1 [34]. 4E-T is directly involved in the repression of AU-rich mRNAs in the cytoplasm [35]; decreased 4E-T levels in the cytoplasm may thus relieve this translational inhibition, modifying B-cell homeostasis. Alternatively, computational analysis suggested that the NES of the cargoes MEK1 would have an increased affinity for CRM1^E571K^. This could lead to a possible blockage of CRM1 and a slowdown in the export of other specific cargoes. The authors also stressed that additional cargoes, such as IKBα and IKBε, with lower difference in affinity between wild-type and mutant CRM1 were found and could also play a significant role in cancer development. Finally, severe mitotic defects were found in the HEK293T CRM1^E571K/E571K^ cells [34]. This supports previous observations describing the recruitment of proteins such as RanGAP and RanBP2 to kinetochores in a CRM1-dependent manner, during the metaphase–anaphase transition [36].

Overexpression of CRM1 is also frequently found in various types of cancers (pancreatic, glioma, ovarian, pulmonary, prostate, and colorectal). It is generally associated with a poor prognosis and resistance to chemotherapies [30,37,38,39]. More precisely, the overexpression of CRM1 has been shown to alter the processes of apoptosis, DNA damage repair, chromosome stability, and angiogenesis [40,41,42,43]. Supporting this idea, a large number of tumor suppressors (such as p53, the retinoblastoma protein RB or BRCA1/2), oncogenic proteins (such as BCR-ABL), and transcription factors (such as FOXO and NF-kB proteins) were found to be mislocalized in cancer cells [24,30,44,45]. 

CRM1 is also considered to be an interesting target in aging pathologies, such as Hutchinson–Gilford progeria syndrome (HGPS), where it is overexpressed [46]. The increase export activity was also observed in the hippocampus and cortex during physiological aging. Here, CRM1 accumulation resulted in a decrease in nuclear TFEB and STAT3 linked with an autophagic flux impairment [47].

### 3.2. Evaluation of the Impact of CRM1 Inhibitors

**Figure 3:**
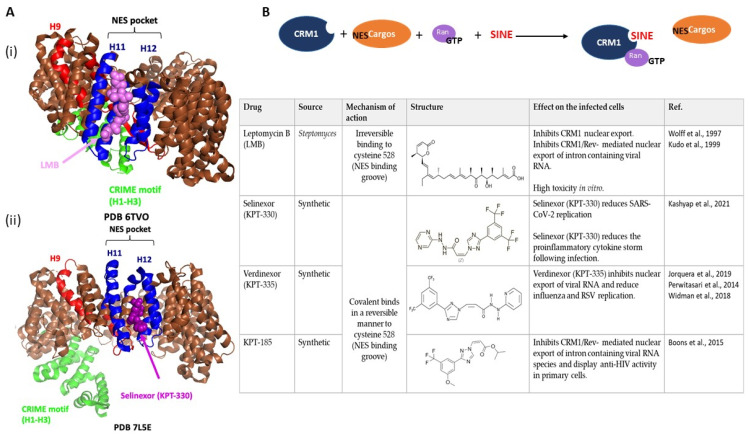
Inhibitors of CRM1. (**A**) Structure of CRM1 in association with SINE. Domains are coloured as descried in Figure 1. (i) Structure of CRM1 in association with leptomycin B (LMB), in purple. RCSB PDB: 6TVO from [61]. (ii) Structure of CRM1 in association with Selinexor (KPT-330), in purple. RCSB PDB: 7L5E from [62]. The two described SINE inhibit CRM1 nuclear export by interaction with the cysteine 528, located on the NES binding groove. All these figures were generate using PyMOL Molecular Graphics System Version 2.5.5. (**B**) Table of the CRM1 inhibitors used in antiviral therapy (see Section 5, Discussion) [6,63,64,65,66,67,68].

A few decades ago, it emerged that excessive nuclear export could contribute to drug resistance during cancer treatment. Many efforts were then expended towards the development of compounds that inhibit CRM1. The latter of these play a critical role in our current knowledge of CRM1-dependent biological processes as well as CRM1-related cargoes. The most well-known inhibitor is leptomycin B (LMB) [48]. Due to its excessive toxicity, a new class of agents was then developed: the “Selective Inhibitors of Nuclear Export” (SINE). These molecules form covalent bonds with CRM1 C528 like LMB (Figure 3) [49]. However, they are engaged in a slowly reversible covalent bonding, which decreases their toxicity [50]. They also cause a transient degradation of CRM1, reversible upon discontinuation of the treatment [51]. Selinexor (KPT-330), the most promising one, is involved in at least 12 phase I and II clinical trials with hematological malignancies and 8 phase I and II clinical trials with solid tumors [52,53,54,55].

Patients with multiple myeloma (MM) commonly acquire a resistance to bortezomid, a proteasomal inhibitor. Quantitative mass spectrometry analysis revealed that CRM1 as well as a strong cluster of XPO1 regulated proteins were upregulated in bortezomid resistant MM cells [56]. In experimental models, the combined treatment of bortezomid with Selinexor improved survival with a decreased tumor development and progression compared with bortezomid alone [57]. Additional studies on gefitinib resistance, ibrutinib resistance, or platinium resistance have demonstrated how reversing CRM1 overexpression through Selinexor inhibition retains key oncogenic signaling proteins and DNA repair factors in the nucleus (β-catenin, DDX17, IκBα, NF-κB p65, p53, SET, Top2A, and E2F) and restores cellular sensitivity to chemotherapy. In addition, in vitro experiments showed that, in macrophages, Selinexor is able to repress the production of TNF-α, a known LPS-induced proinflammatory cytokine, notably through suppressing NF-kB signaling. Notably, CRM1 inhibition induced the nuclear retention of p65 and IκBα, retaining NF-κB subunits in a repressed complex. As a consequence, the NF-κB regulation and activation pathways were disrupted, reversing its proinflammatory profile [58]. Similarly, the use of the SINEs KPT-350 and KPT-335 showed an upregulation of anti-inflammatory cytokines (IL-4, IL-10 and IL-13) and a downregulation of proinflammatory cytokines (including IL-1A/B, IL-6 and IL-8) revealing the central role of CRM1 in the inflammatory response [59,60].

### 3.3. Investigatation of CRM1 Interactome

Finally, to investigate the biological pathways involving CRM1, the CRM1 interactome was studied. In 2020, Miloudi et al. compared the interactome of WT and E571K CRM1 [33], while Kirli et al. had previously compared the interactome of CRM1 in *S. cerevisiae*, in *Xenopus* oocytes and in HeLa cells [69]. In this latter study, they found more than 700 cargoes in yeast, 1000 cargoes in *Xenopus* oocytes, and >1050 cargoes in HeLa cells. These results enrich the existing data concerning the link between CRM1 and the biogenesis of ribosomes, the export of regulatory proteins such as kinases, phosphatase, and the ubiquitin/proteasome system, autophagy, the regulation of centrosomes; they also highlight new links with vesicular assembly of secretory pathways, peroxisomes, and RNA metabolism. Interestingly, among the high-scoring cargoes, they identify factors involved in mRNA degradation, as well as components of P-bodies, suggesting a potential regulation of RNA metabolism. These cargoes are also found in *Xenopus* oocytes and *S. cerevisiae*, suggesting a conserved function of CRM1 [69]. 

mRNA export is a critical step of gene expression that is modulated by the incorporation of nuclear export adaptors in the mRNP at different steps of the RNA maturation [70]. For bulk mRNA, the heterodimer NXF1/NXT1 ultimately binds RNA and these adaptors in a cooperative manner to drive nuclear export through the NPC. However, for a subset of RNAs, the transit through the nuclear pore is dependent on the CRM1/RanGTP complex, instead. To do so, CRM1 relies on specific RNA-binding adaptors displaying NES. So far, four of them have been clearly identified: HuR (RNA-binding protein human antigen R), LRPPRC (leucine-rich pentatricopeptide repeat protein), NXF3 (nuclear export factor 3), and PHAX (phosphorylated adapter for RNA export). HuR binds mRNAs containing AU-rich elements (AREs): these AREs are found in the 3′UTR region of up to 22.4% of human mRNAs, including most inflammatory cytokines, interferons, and other transiently expressed chemokines genes [71,72,73]. On this topic, CRM1 is the nuclear export receptor required for the export of *c-fos* and *Cox2* mRNA [73,74]. Direct evidence has also been reported for *TNF-α*, *BIRC3*, *LTA*, *CD82*, *NINJ1*, *BCL2L1*, and *CD83* mRNA in activated lymphocytes specifically [75]. LRPPRC, in association with eIF4E, controls the export of mRNAs with 4E-SE sequences in their 3’UTR [76,77]. This eIF4E–4ESE-dependent export is involved in the regulation of cell proliferation, in particular with the export of *cyclin D1* mRNA [78,79]. CRM1 interacts with NXF3 to export small nucleolar RNA (snoRNA) already associated with a large multiprotein complex [80] in response to stress conditions [81], as well as piRNA precursors (PIWI-interacting RNA small non-coding repressor RNAs) in Drosophila [82]. Interestingly, these adaptors of export are overexpressed in cancer cells, like CRM1, strengthening a particular link between mRNA export, CRM1 and oncogenesis [37]. Finally, CRM1-RanGTP interacts with PHAX to export small nuclear RNA (snRNA) precursors that are involved in mRNA splicing and rRNA and histone preRNA processing after maturation [5,83]. These precursors are hypermethylated in cytoplasm by a methylase exported by CRM1 and TGS1, as shown through a heterokaryon assay [84,85]. The characterization of CRM1 functions associated with snoRNA metabolism also reveals some export-independent functions; indeed, CRM1 can orchestrate pre-snoRNP remodeling and promote their nucleolar targeting: by removing the long isoform of TGS1 bound to the snoRNP, CRM1 activates the NoLS of the core snoRNP protein NOP58, leading to its nucleolar relocalization [86,87].

A number of other mRNAs, such as *Interferon-alpha-1 (IFN-α1)* and *interleukin 6 (IL-6)*, are exported by CRM1, although no adapter is currently known [88,89]. *IFN-α1* is expressed quickly after infection and plays a key role in innate defense against pathogens and almost every aspect of cellular and humoral adaptive immune responses. Similarly, *IL-6* is a pleiotropic cytokine with a central role in the integrated immune defense network against infection. IFN-α1 transcripts are exported in a CRM1-dependent and HuR-independent manner and would rely on secondary structures present in the coding sequence [90,91]. Concerning *IL-6* mRNA, the inhibition of its export by LMB treatment suggested the involvement of CRM1. After LPS stimulation, Arid5a (adenine-thymine (AT)-rich interactive domain 5a) binds to the stem loop structure in the 3′UTR of *IL-6* mRNA and is exported with *IL-6* mRNA. The absence of an identified NES in Arid5a implied the existence of another adapter. Interestingly, the authors observed that the RNA helicase UPF1, known as a CRM1 cargo, was exported with Arid5a/*IL-6* mRNA as well [92]. However, proposing UPF1 as a new CRM1 adapter would require further investigation.

## 4. Viral Subversion of the CRM1 Function

**Figure 4:**
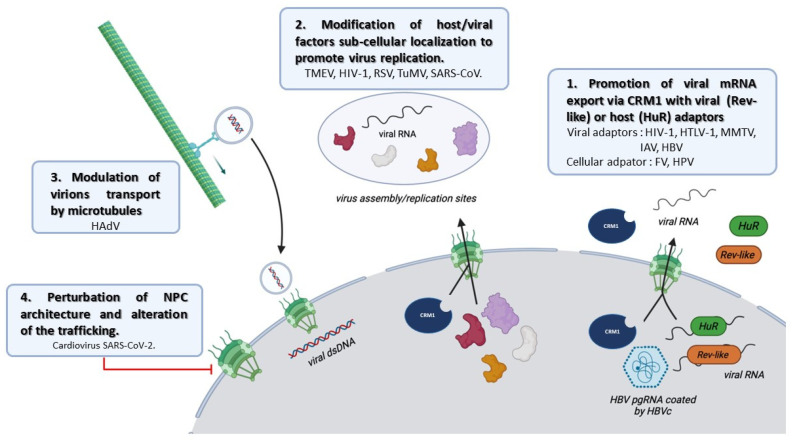
Scheme summarizing the consequences of the viral hijacking of CRM1. (1) Upon viral infection, it is well described that CRM1 is hijacked to support vRNA export with a viral NES adaptor or with HuR. (2) The re-localization of cellular proteins is also described as a consequence of CRM1 hijacking, notably to the site of virus assembly or/and virus replication sites. (3) In HAdV, CRM1 plays a crucial role by enhancing the microtubule mediated transport of the virions to the nuclear membrane. (4) In some cases, CRM1 hijacking is involved in the alteration of the NPC composition, affecting the nucleocytoplasmic trafficking.

As described before, CRM1 is a central factor controlling the flux between nucleus and cytoplasm. Viruses have developed strategies to exploit the cellular machinery, including the CRM1-dependent export, to allow replication and transmission. Among the studies reporting a viral hijacking of CRM1, we identified four main functions for CRM1 during the viral replication cycle (Figure 4): First, the selective export of viral RNA that relies on specific RNA-binding adaptors and allows the bypass of the canonical export pathway. Second, the adaptation of the cellular transport network to favor the viral components mobility and virus assembly. Only few examples have been reported so far, but they match with the function of CRM1 related to the organization of the cytoskeleton and centrosome. Third, the redistribution of host factors to promote viral replication; by interacting with CRM1, viral proteins are able to contact and control specific host proteins shuttling, redirecting them toward proviral activity. Fourth, several examples in the literature have described how viruses exploit CRM1 to access the NPC in order to modulate its composition, altering nucleocytoplasmic trafficking.

### 4.1. Viral Interference with the CRM1 Export Pathway

#### 4.1.1. Drive of vRNA Export

Retroviral genomic information is contained in a single RNA that is reverse transcribed into DNA. This proviral DNA is then integrated in the host genome. In order to replicate, the complete proviral DNA sequence is transcribed, and the primary transcript (full length, vRNA) undergoes various splicing events. Viral RNAs are exported for protein synthesis. Notably, the unspliced viral RNAs (vRNAs) usually code structural proteins that have to be expressed in higher amounts. In addition, vRNA has to be encapsidated as is, to form mature and infectious viral particles. Thus, viral RNAs have to be efficiently exported and also need to avoid surveillance mechanisms that basically prevent the export of unspliced mRNA. To overcome these constraints, complex retroviruses bypass the usual NXF1/NXT1 mRNA export pathway and use the CRM1 export pathway, as shown for HIV-1 [6]. Most of the complex retroviruses can be characterized by the expression of an adapter protein dedicated to connect CRM1 and vRNA. Historically, the discovery of the viral hijacking of CRM1 by the retroviral protein Rev from HIV-1, played a key role in the deciphering of CRM1 mode of action and functions [7]. First, Rev binds RNA at the Rev-responsive element (RRE) in the middle of the Env coding exon [6,93]. The RRE is a highly structured RNA element of ~350-nt containing multiple binding sites for Rev (reviewed in [94]). It was shown that multiple molecules of Rev are recruited on the RRE in a specific and cooperative manner [95]. Second, Rev displays a leucine-rich sequence fitting the NES consensus and recognized by the exportin CRM1 [9,96]. Both proteins associate prior to or during early spliceosome assembly at the transcription sites. Third, the local concentration of Rev multimers stabilizes the Rev–CRM1 interaction and is correlated with viral mRNA export efficiency [97]. Like snRNA or snoRNA, unspliced or partially spliced HIV-1 mRNA harbor a trimethylguanosine (TMG)-cap catalyzed by the human methylase, PIMT (paralog of the yeast TGS1) in a Rev-dependent manner. The TMG-cap likely serves to direct these RNA to CRM1 export; it also inhibits cap exchange to eIF4E, favoring the specialized translation of HIV late mRNA [98,99]. 

Other complex retroviruses express Rev-like proteins and mimic this process; for instance, the Rem and Rex proteins from the betaretrovirus MMTV and the deltaretrovirus HTLV-1, respectively. Rex (the most-described retroviral adapter after Rev) displays NES, NLS and a dual multimerization domains like Rev, despite a globally low sequence homology with Rev (reviewed in [100]). While the overall mechanism of vRNA export is similar to that which is described for Rev, some divergences have been characterized: notably, it is accepted that Rex can interact with CRM1 as a monomer before multimerization and RNA [101,102]. The functional homology of these proteins even allowed the successful complementation of Rev- and Rem-deficient viruses with Rex [103]. Along the same lines, the Orthomyxoviridae influenza A virus (IAV –) is an RNA virus using CRM1 to export some of its RNA. The IAV nucleoprotein (IAV NP) coating vRNA to form vRNPs, accumulates in the nucleus after LMB treatments and in the cytoplasm after CRM1 overexpression [104,105]. In parallel, the viral nuclear export protein (NEP/NS2) and matrix (M1) protein also display several NES sequences, supporting a model in which vRNPs are exported in conjunction with NP, M1, and NEP. In this daisy-chain model, M1 acts as a bridge between the NP bound to RNA and NEP, while NEP is an adapter contacting CRM1 [106,107,108]. This RNA–M1–NEP–CRM1 complex is reminiscent of the jelly fish model described for Rev complexes. 

Interestingly, foamy viruses (FVs) are complex retrovirus that do not encode Rev-like proteins to support vRNA export. FVs take advantage of the cell adapter, HuR, combined with ANP32A/B [109]. Crosslinking experiments revealed that the host protein HuR is co-recruited on the viral mRNP together with CRM1, but the targeted RNA sequence remains to be identified. Likewise, human Papillomaviruses (HPVs) late genes mRNA (L1 and L2) contain well defined AU-rich inhibitory elements, named h1ARE [110,111,112,113]. HuR binds these sequences and stimulates HPV-1 late gene expression in a CRM1-dependent pathway [114].

At last, some viruses hijack CRM1 to segregate subpopulations of the same RNA. The simple retrovirus Mason–Pfizer Monkey Virus (MPMV) illustrates that point: MPMV has well characterized cis-elements, called constitutive elements (CTEs), dedicated to the NXT1/NXF1 export. However, Mougel et al. showed that the RNA export pathway involved is dependent on the future of the vRNA molecule (translation or encapsidation); RNA imaging and export reporter assays demonstrated that NXF/NXT1 drives vRNA for translation, while the CRM1 pathway marks the MPMV RNA for packaging [115]. Naturally, the Psi sequence for packaging is critical in this process but the adapter is still unknown. Similarly, the Herpes virus hijacks CRM1 to selectively export its RNA. Illustrating this, HSV-1 has a nuclear replication cycle and needs to export vRNA in the cytoplasm for translation. It encodes ICP27 (infected cell culture protein 27), a shuttling RNA binding protein, that affects pre-mRNA processing. ICP27 is described to facilitate viral mRNA export via the RNA export REF and export receptor NXF1 [116,117]. However, it has also been reported that ICP27 contains a NES [118,119,120]. Soliman et al. demonstrated that the mutagenesis of ICP27 NES domain affects the virus growth and that LMB prevents ICP27 and vRNA cytoplasmic accumulation [121,122]. Thus, similarly to MPMV, HSV-1 exports some viral mRNAs using CRM1-independent pathway while other viral RNA are exported via a CRM1-dependent pathway. For adenovirus type5, it is admitted that the NXF1 complex is needed for viral late mRNA export [123], while one study showed that CRM1 is involved in viral early mRNA export [124], also illustrating a selective use of export pathways during infection.

Hepatitis virus (HBV) is another example of DNA virus that seem to use CRM1 to export encapsidated viral RNA (pgRNA). Yang et al. showed that when HBV core protein (HBc) assembles as a viral particle in the nucleus, it has multiple accessible NES domains. Those viral particles are filled with pgRNA and exported by CRM1 in the cytoplasm for genome maturation. CRM1 inhibitors treatments then significantly inhibit HBc export leading to a reduction in viral DNA synthesis. This alternative model of pgRNA encapsidation would occur in the early phases of infection, to the contrary of the late phase, when pgRNA export is NXF1-dependent [125]. This illustrates how the hijacking of CRM1 tightly controls vRNA export.

#### 4.1.2. To Adapt the Transport Network to Favor Viral Components Mobility and Virus Assembly

Clear evidence exists to demonstrate how adenoviruses take advantage of CRM1 to precisely drive virions from cytoplasm to the nuclear membrane [126]. By combining high-resolution microscopy, live imaging, and machine learning, Wang et al. found that CRM1 inhibition increases the fraction of microtubule-dependent motion across the cell, preventing the unloading of virions around the nucleus [127]. It is suggested that CRM1 may bring a factor such as HDAC or AGTPB1/CCP1 to the nucleus proximal microtubules that could control the interaction between microtubules and motors. Ultimately, this would reduce the transport of cargoes such as adenoparticles and further deliver their genome in the nucleus. Recently, Lagadec et al. identified two new mutations CRM1 W142A and P143A that are compromised with respect to adenoviral capsid disassembly in both interphase and mitotic cells but remains export competent [128]. This revealed that viruses can take advantage of export-independent functions of CRM1 during viral infection. 

#### 4.1.3. To Control Host Factors Redistribution Promoting Viral Replication

As described before, CRM1 controls the localization of numerous proteins within the cell. Naturally, those factors are also important regulators of the viral replication and are often redistributed to favor specific steps of the viral life cycle. In line with this, several studies suggested that CRM1 hijacking could help forming a hub to come in contact or to better control the export of these nuclear partners of CRM1. Interestingly, this process is observed with viruses replicating in the nucleus as well as in the cytoplasm. 

As discussed before, retroviruses hijack CMR1 with Rev-like proteins in the host nucleus to export vRNA. It is important to note that several other host proteins have been identified with this CRM1-Rev like-vRNA complex. For instance, the RNA helicase UPF1, STAU2, the RNA binding motif protein 14, eukaryotic initiation factor-5A, and the DEAD box RNA helicases DDX1, DDX3, and DDX5 are recruited on the vRNP with CRM1 and stimulate nuclear export [129,130,131,132,133,134,135,136,137,138,139]. Some of these factors, such as UPF1, were shown to exert additional proviral activities when associated with the vRNP: in HIV, it was suggested that UPF1 is incorporated in the viral particle to support reverse transcription functions in a newly infected cell [140]. In line with this, Prochasson et al. recently observed that the REX–CRM1 interaction is critical for the incorporation of UPF1 in the HTLV mRNP [129]. In the late steps of HTLV-1 replication, UPF1 is necessary for optimal RNA export, to control the maturation of structural proteins, and ultimately, to produce infectious virions.

The turnip mosaic virus (TuMV) is an ssRNA(+) virus whose replication occurs in cytoplasmic bound membrane complexes (viral replication centers—VRCs), adjacent to the nucleus. These complexes are composed of host factors as well as several viral proteins including the NIb protein. NIb is the viral RNA polymerase. It harbors 2 NES sequences in the RdRP domain and interacts with CRM1 [141]. Upon sumoylation, CRM1 exports NIb out of the nucleus where both factors participate in the formation of the VRCs [142]. The exact composition of the CRM1–NIb complex has not yet been investigated but may reveal the presence of numerous host factors also found at the VRC. Those results demonstrate that the interaction of viral protein with CRM1 also plays a critical role in the replication of plant virus. 

The matrix protein (M) from the respiratory syncytial virus (RSV), an ssRNA(−) virus, is exported in a CRM-dependent manner to promote virions production [143]. Indeed, residues 194–206 from the RSV M protein fit the consensus to a NES. Likely favoring the interaction with CRM1 NES pocket, the phosphorylation of Threonine 205 by CK2 enhances M nuclear export. It is tempting to link the shuttling of M with an already known function of M in mediating the transport of vRNPs to assembly sites; although a direct connection with the known actin-driven transport by M has yet to be investigated, we may suspect this viral protein to redirect CRM1 functions associated with intracellular transport: indeed interactome studies highlighted a connection of CRM1 with cytoskeleton organization through interactions with septins and tubulin, as well as microtubule organization centers [60].

#### 4.1.4. To Alter the Nuclear Pore Composition

Following the idea to control host factors localization, a large number of viruses directly target the Nups of the NPC. They act on the NPC permeability and affect the nucleocytoplasmic trafficking and to favor viral replication.

It was recently reported that CRM1 is concentrating host factors necessary for SARS-CoV-2 replication. In parallel, at least 7 SARS-CoV-2 viral proteins display a putative NES (Table 1). More specifically, it was observed that CRM1 directly affected the interaction between ORF6 from SARS-CoV-2 and NPC components like Rae1 or Nup98 [144,145]. This interaction with Rae1 and Nup98 is associated with mRNA accumulation the nucleus.

It also has been described and documented in detail how viral proteases could cleave the FG domains of Nups to open the pore. However, in these cases, there is no evidence of CRM1 involvement. Interestingly, the Cardioviruses (Picornaviridae) such as EMCV and TMEV, whose proteases are inactive, use the leader protein, L, to induce the hyperphosphorylation of NUP62/NUP153/NUP214 and Nup98 by specific kinases, such as ERK, RSK, and p38, to alter the pore functioning. Concomitantly, L was shown to interact with Ran and CRM1; size exclusion experiments suggested that a trimeric complex exists and CRM1 knockdown strongly diminished NUP62 phosphorylation in EMCV-infected cells [146,147]. Altogether, these data support the model that L drives the hyperphosphorylation of Nups by interacting with Ran, CRM1, and specific kinases [148]. In the case of picornaviruses, these alterations are likely to favor the recruitment of nuclear proteins in the cytoplasm to promote viral replication and/or translation; notably, it is reported that TMEV L protein induces the cytoplasmic mis localization of polypyrimidine tract binding (PTB), known to be essential for TMEV IRES-dependent translation initiation [149].

**Table 1:** List of described viral proteins (viral family/virus name/viral proteins) exported/interacting via CRM1. When known, the NES sequence is indicated. NetNES1.1 is an online tool to predict NES (http://www.cbs.dtu.dk/, accessed on 1 July 2023). Hydrophobic acids are colored in red and when the sequence fit the consensus, the class (from [13]) is determined. The known functions/consequences of viral CRM1 hijacking on the host cell the host cell are briefly summarized.

Type of Genome	Family	Virus Name	Viral Protein	NES Sequence Hydrophobic Acids Are in Red	Effect of Nuclear Export on the Host Cell and/or Virus Cycle	References
ssRNA(+)	Retroviridae	Human immunodeficiency virus type 1 (HIV-1)	Rev	LQLPPLERLTLD Class 1b	Nuclear export of Rev is essential to support unspliced viral RNA export from nucleus.	[6,7]
			Vpr	EAIIRILQQLLFI Class 1a-R	Nuclear export of Vpr is required for efficient replication.	[150]
		Human T-cell leukemia virus type 1 (HTLV-1)	Tax	YKRIEELLYKISLTT Class 1a	N.D	[151]
			Rex	ALSAQLYSSLSLDS Class 1c	Support CRM1-dependent export of the unspliced viral RNA.	[101,152]
			HBz	MVNFVSVGLFRCLPVPCPEDLLVEELVDGLLSL	Nuclear export of HBZ is essential to regulate the mTOR signaling pathway via inhibition of GADD34 activity in the cytoplasm.	[153]
		Mouse mammary tumor virus (MMTV)	Rem	N.D	Support CRM1-dependent export of the unspliced viral RNA.	[154]
	Coronaviridae	SARS-CoV-2	ORF9	LALLSDLQDL Class 1b *(putative NES)*	N.D	NetNES 1.1
			N (Nucleocapsid)	LLDRLNQL Class 1a *(putative NES)*		
			S (Spike)	LEPLVDLPI Class 1b *(putative NES)*		
			M (Matrix)	LESELVIGAVIL Class 1c *(putative NES)*		
			E (Enveloppe)	LAILTALRL Class 1b *(putative NES)*		
			ORF3a	VHFVCNLLL Class 1b *(putative NES)*		
			NSP12 (RdRp)	LMIERFVSLAI Class 1b (putative NES)		
			ORF6	N.D	By interacting with Nup98/Ra1, ORF6 inhibits CRM1-dependent export of IRF-1 and RIG-I mRNA.	[155]
		SARS-CoV	ORF3b	L-HKLLQTLVL Class 1c	Suppression of IFN I signaling (mitochondrial antiviral signaling—MAVS)	[156]
			ORF9b	LRLGSQLSL Class 2	Inhibition of apoptosis caspase-3 dependent.	[157]
	Alphaviridae	Venezuelan Equine Encephalitis (VEE)	Capsid	TDPFLAMQVQELTRSMANLTFKQRRDAPPEGPSAKKPKK	N.D	[158]
			nsP2	VREFGLDLDSGL Class 4	N.D	[159]
	Flaviviridae	Hepatitis C virus (HCV)	Core protein	LGKVIDTL Class 1d	N.D	[160]
		Dengue virus (DENV)	NS5 (RNA polymerase)	LLTKPWDIIPMVTQMAM Class 1a	Modulation of IL-8 production	[161]
		Zika virus (ZIKV)	NS3 (protease)	TRVVAAEMEEALRGL Class 2	N.D	[162]
		Chikungunya virus (CHIKV)	Capsid	VKGTIDNADLAKLAF Class 1a	N.D	[163]
	Potyviridae	Turnip Mosaic Virus (TuMV)	TuMV Nlb (RNA Polymerase)	YEYWWDTWADNWREW	Interaction between Nlb and CRM1 required to promote viral replication	[141]
	Picornaviridae	Cardiovirus	L protein	N.D	Alteration of nuclear pore complex homeostasis.	[147]
		Mengovirus	L protein	N.D		[164]
ssRNA(−)	Bornaviridae	Borna disease virus (BDV)	X protein	LRLTLLELVRRL Class 2	Nuclear export of X protein is essential to mitochondrial localization of the viral protein. Mitochondrial localization required to its neuroprotective activity.	[165]
	Orthomyxoviridae	Human parainfluenza 2 virus (HPIV-2)	P (Phosphoprotein)	IIELLKGLDL Class 1d	N.D	[166]
		Influenza A (IAV)	NP (Nucleoprotein)	Several sequences: MIDGIGRFYI Class 1b VKGVGTMVM Class 1b LIFLARSALIL	Support viral vRNP export.	[104]
			NS2/NEP (Nuclear export Protein)	MITQFESAKA Class 1b		[167]
			M1 (Matrix)	ILGFVFTLTV Class 1b		[108]
		Influenza D (IDV)	NS2	LVSLIRLKSKL Class 1d		[168]
	Pneumoviridae	Respiratory Syncytial Virus (RSV)	M (Matrix protein)	IIPYSGLLLVITV	Support virus particle formation and vRNA export. CRM1-mediated nuclear export is critical to the infectious cycle of RSV.	[143]
dsDNA	Herpesviridae	Cytomegalovirus (CMV)	Pp65 (Matrix protein)	NLVPMVATV	Support viral RNA export. Pp65 export is important for pp65 antigen presentation and CHD activation.	[169]
			UL84	Two independent NES: LSLNLFALRI LTLSSLTL Class 2	Support viral RNA export.	[170]
			UL94	CILCQLLLLY	N.D	[171]
		Kaposi’s sarcoma-associated herpesvirus (KHSV)	LANA 2	MVP-LVIK-LRL Class 2	Subcellular localization of LANA2 regulate p53 activity and apoptosis induction. LANA2 export regulate lytic viral cycle.	[172,173]
			ORF45	VLSQRIGLMDV	N.D	[174]
		Herpes simplex virus type 1 (HSV-1)	pUL47 (Tegument protein)	IMSQFRKLLM Class 1b	pUL47 binds to RNA transcripts.	[175]
			ICP27	DMLIDLGLDLDL Class 2	Export of ICP27 is crucial for viral production by playing a role in ICP4 expression, the mRNA export factor.	[121,176,177]
			ICP34.5	LPPRLALRLR	N.D	[178]
		Bovine herpesvirus type-1 (BHV-1)	BICP27 (Bovin ICP27)	LEELCAARRLSL	N.D	[179]
	Adenoviridae	Human Adenovirus tye-5 (HAdV-5)	E4	MV--LTREELVI Class 1c	N.D	[180]
			E1A	VSQIFPDSVMLAVQEGIDLL Class 1b	N.D	[181]
			E4orf6	N.D	E4 is co-exported from nucleus with the complex HuR-mRNA (*c-fos*, *c-myc*, *COX-2*), increasing mRNA stability and inducing cell transformation.	[182,183]
	Hepadnaviridae	Hepatite B virus (HBV)	HBx (Protein X)	QILPKVLHKRTLGLSAM	NES domain of HBx is associate with cytoplasmic retention of CRM1.	[184]
			HBc (Core protein)	Two sequences: WGELMTLATWVGNL Class 1a-R RDLVVSYVNTNMGL Class 1c or 1d	Interaction with CRM1 allowed HBC particles containing encapsidated viral RNAs.	[125]
	Papillomaviridae	Human papillomavirus type 11 (HPV 11)	E1	ISPRLDAIKL Class 1a	Nuclear export of E1 is necessary for long-term viral DNA maintenance and cell proliferation.	[185]
			E7	IRQLQDLLL Class 1b	N.D	[186]
		Human papillomavirus type 16 (HPV 16)	E7	IRTLEDLLM Class 1b	N.D	[187]
		Human papillomavirus type 8 (HPV 8)	E7	IRTFQELL Class 3	N.D	[188]
ssDNA	Parvoviridae	Murine minute virus (MVM)	NS2-P (non-structural protein 2)	MTKKFGTLTIHDTEKYASQPELCNN	Nuclear export of NS2-P is essential for proper viral production.	[18,189]
	Anelloviridae	Chicken anemia virus (CAV)	VP1 (Nucleocapsid)	ELDTNFFTLYVAQG Class 1c-R	N.D	[190]

Finally, the Hepatitis C virus (HCV), an ssRNA(+) with a cytoplasmic replication cycle, expresses a core protein (HCVc) that is localized in the cytoplasm and in the nucleus [159]. The domain responsible for an interaction with CRM1 is well-characterized and LMB treatments induced a nuclear retention of HCVc. Although LMB treatments in an HCV replicative cellular model also showed a decrease in the production and release of new viral particles, the underlying molecular mechanisms are yet to be clarified. It is possible that thanks to this shuttling, HCVc captures nuclear factors necessary for late steps of the infection or induces their re-localization. Supporting this, Boson et al. recently showed that the NPC component Nup98 is recruited by HCVc to viral assembly sites, a necessary step to locally increase the concentration of (+) RNA genome [191]. The common interaction of HCVc and CRM1 with Nup98 combined with the CRM1/HCVc association might help targeting the HCVc to Nup98. 

### 4.2. Viral Interference of CRM1 to Modulate Cellular Pathways

As described previously, viruses exploit CRM1 in different steps of the viral replication, notably to support viral RNP export or to re-localize host factors to promote viral activities. There is much less evidence supporting that this hijacking, by competing with cellular cargoes, may also alter host biological processes. However, we know that the innate immune system activates signaling cascades that lead to the nuclear localization of interferon (IFN)-regulatory factors, such as IRF-3/7, NF-kB (p50/p65), STAT, and /or cGas to stimulate the expression of type I interferon and proinflammatory cytokines [192,193,194,195]. To maintain their signaling cascades actives, these regulators need to be exported back in the cytoplasm by CRM1. We also described above how CRM1 favors the cytoplasmic export and stabilization of *IFN-I* and *cytokines* mRNA, including *IL-8*, *IL-6*, *TNF-α*, *COX-2*, and many others. Here, we report the different studies that revealed a direct or indirect impact of CRM1 hijacking on the pathological phenotypes during infection.

#### 4.2.1. Modulation of Immune and Antiviral Signaling

In cells overexpressing HIV-1 Rev, it has been monitored by ELISA that Rev reduced the nuclear export of human interferon-α1 (IFN-α1) [91]. This inhibition was reversed when Rev had a point mutation in its NES, suggesting that this effect on host mRNA export is the consequence of a competition between CRM1-dependent export for viral RNA and cellular mRNA and is consistent with the observation that CRM1 and Ran is a rate-limiting step in nuclear export. Along the same lines, the recruitment of SARS-CoV-2 ORF6 to the nuclear pore by CRM1 was shown to inhibit the export of many mRNA including those encoding antivirals factors such as IRF-1 and RIG-I, leading to the suppression of the antiviral response [155]. 

HBx protein is a known inhibitor of CRM1, as described before. Forgues et al. reported that its expression clearly modified the localization of NF-κB, inducing its nuclear retention, while HBx itself was essentially cytoplasmic. NES-mutated HBx was retained in the nucleus and the usual nucleocytoplasmic distribution of NF-κB was maintained. Thus, the hijacking of CRM1 seems to be directly involved in the deregulation of NF-κB. However, Forgues et al. found that this nuclear accumulation was associated with a NF-κB activation, while CRM1 inhibitors induced a nuclear retention of Iκ-Bα in the nucleus and p65 in a repressed complex, disrupting NF-κB signaling [184,196]. Lim et al. resolved this apparent paradox with the identification of a nuclear ternary complex composed of HBx/p22Flip/NEMO responsible for NF-κB activation [197]. This suggests that, despite HBx exerting multiple effects to maintain NF-κB activation, CRM1 hijacking by HBx clearly has a repressive action on NF-κB. 

Several cytoplasmic-replicating RNA viruses harbor an intermediate of dsRNA during the life cycle. dsRNA is recognized as a PAMP and activates innate antiviral signaling. Indeed, this cytoplasmic dsRNA from pathogens are detected by retinoic-acid-inductile gene I (RIG-1). The mitochondrial antiviral signaling (MAVS) activates IRF-3 or NF-κB (p65/p30) and promotes their translocation to produce IFN-I and cytokines. SARS-CoV is threatened by this pathway. Freundt et al. observed, through confocal microscopy, that the SARS-CoV ORF3b protein has nuclear, cytoplasmic, and mitochondria localization in the cell. LMB treatments induce a nuclear retention of ORF3b and a reduction in mitochondria localization, suggesting that the CRM1-dependent export of ORF3b is needed for its mitochondrial targeting. By using an IRF3-inducible luciferase reporter assay, they found a decreased level of the IRF3-inducible reporter when ORF3b is located on mitochondria. In summary, SARS-CoV ORF3b is exported from the nucleus to the mitochondria via CRM1, leading to MAVS signaling inhibition [156].

#### 4.2.2. Modulation of Pro-Oncogenic Cell Activity

In addition to subvert the host immune signaling, viruses such as oncovirus (but not exclusively) can specifically take control of the host pathways dedicated to proliferation and survival/apoptosis. Likewise, we reported before how CRM1 mutagenesis or overexpression was associated with cancer and recent studies have proposed that CRM1 cargoes are involved in 9 out of 10 cancer hallmark processes [42]. In 2019, Guven-maiorov et al. developed a computational method to predict host/microbes’ interactions (HMI) based on exogenous surface mimicking. With this tool, they analyze the HMIs of all oncoviral proteins with available 3D structure and investigated their impact on the cancer hallmark traits [198]. Even though the results were under-evaluated because of the lacking available structures of viral proteins, CRM1 was suggested as a target for several proteins of more than half of the oncovirus. This convergence, combined with the central place of CRM1 in non-viral-induced cancers, suggests that CRM1 alteration during infection may deregulate the signaling of some cancer hallmark process. However, few studies were interested in this aspect. 

We can report the case of the oncogenic HBx (Hepatitis B) leading to hepatocytes transformation, during HBV infection. The oncoprotein HBx contains a functional NES domain that is necessary for its cytoplasmic localization [184]. The authors observed a cytoplasmic sequestration of CRM1 in HBx-overexpressing cells as well as in infected livers. This example is one of the rare that illustrates how a viral protein can force an overall abnormal localization of the exportin. Interestingly, they also observed centrosomal duplications that were directly dependent on CRM1 sequestration and inactivation by HBx [199]. Abnormal centrosomal duplications is a hallmark of cancer [200]. Mechanistically, these results fit with the role of CRM1 in regulating the formation of a bipolar spindle and centrosome [201]. Recently, Shi et al. found that HBx interacts with Smad4 and prevent the nuclear translocation of Smad2/3/4 leading to deprive activin antiproliferative activity. Indeed, as a part of activin signaling, Smad proteins works as an inhibitor of cell growth. This effect is CRM1-dependent and reversed upon LMB treatment. HBx, while inducing the cytoplasmic relocation of CRM1, prevents the continuous shuttling of these proteins necessary for their action. Although the exact underlying mechanism is still unknown, this illustrates the critical influence of CRM1 hijacking by a viral factor in the destabilization of the host homeostasis [202]. We reported before how HBx interaction with CRM1 also affects NF-κB activation. NF-κB is well known to control the expression of target genes, involved in tumor cell proliferation, survival, and angiogenesis; in line with this, NF-κB deregulation via the HBx/CRM1 axis could exert oncogenic effects. 

It is noteworthy that examples also exist of an indirect alteration of CRM1 export by viral proteins inducing a modulation of oncogenic ARE-mRNA expression. This is the case of the adenovirus (dsDNA virus) E4orf6 protein that is not exported by CRM1, but displays a putative NES at its N-terminal extremity [203]. Higashino et al. demonstrated on transformed BRK cell lines that the N-terminal extremity of E4orf6 is responsible of a nuclear retention of pp32/LANP protein, involved in the HuR-mediated export of ARE-mRNA *c-fos*, *c-myc*, and COX-2. In addition, the oncodomain on the C-terminal extremity of E4orf6 can interact with pp32/LANP and restore its distribution as well as mRNA export after complexing with E1B-55K and HuR. However, this was possible even under LMB treatments, demonstrating that E4orf6 can inhibit CRM1 and favor a CRM1-independent export of ARE-mRNA, which are critical in the transformation of rodent immortalized cells [182]. Finally, Dobner’s group identified and characterized adenoviral E1B-55K shuttling via CRM1. Notably, they showed that E1B-55K SUMOylation, close to the NES, interferes with CRM1 binding. This leads to E1B-55K accumulation at the nuclear periphery, close to viral replication centers of infected cells and PML nuclear bodies in stably transformed rat cells. SUMO deconjugation then restores a competent CRM1-dependent export [204]. Interestingly, among E1B-55K many host interactors, p53 and Mre11 are exported in the cytoplasm for proteosomal degradation: this nucleocytoplasmic export and degradation by the E1B-55K/E4orf6-dependent E3 ubiquitin ligase was somehow affected by mutations of E1B-55K NES [205]. In transformed rat cells, this mutant exhibited increased tumorigenicity, indicating that E1B-55K-CRM1 interaction also controls oncogenic transformation by combinatorial mechanisms that involve the modulation of p53 in the context of PML nuclear bodies [206].

## 5. Discussion

In this review, we exhaustively listed viruses and viral proteins that contain NES. This reflects their need to frequently navigate between the nucleus and the cytoplasm. Among the many examples that we present here, it is clear that, beyond their cytoplasmic localization, the transit through the nucleus is essential to ensure efficient viral replication. It appears that CRM1 hijacking allows the shuttling of different types of viral materials, such as viral proteins and viral mRNPs, as well as allowing the formation of complex viral assemblies such as viral particles. Viruses also take advantage of the central place of CRM1 in the cell and its important interactome, to hijack or inhibit host cargoes. Surprisingly, the impact of CRM1 hijacking on cellular processes is much less documented. However, it seems that the export of RNAs encoding cytokines and interferon is altered in this process.

In line with these observations, CRM1 inhibitors (SINEs) were recently used as antiviral treatments (Figure 3); the SINE compound KPT-185 suppressed the expression of the intron containing late viral RNA species, displaying anti-HIV activity in primary cells [66]. Similarly, Verdinexor (KPT-335) administration showed an antiviral activity at tolerable dose range for dsDNA opportunistic viruses such as Epstein–Barr virus (EBV), human cytomegalovirus (HCMV), Kaposi’s sarcoma virus (KSHV), adenoviruses (AdV), BK virus (BKPyV), John Cunningham virus (JCPyV), and human papillomavirus (HPV), in vitro [65]. Strikingly, the team of Ralph. A. Tripp found that Verdinexor treatments could repress not only viral replication but also the proinflammatory cytokine production after RSV and IAV infections [63,64,207]. This was associated with a decrease in the viral dependent lung inflammation and the mortality rate in animal models. The capacity of CRM1 inhibitors to favor anti-inflammatory cytokines and downregulate pro-inflammatory cytokines production thus reduced the virus-associated immunopathology. In view of these observations on respiratory RNA virus, SARS-CoV-2 infection models were treated with Selinexor in vitro and in vivo. Kashyap et al. found that XPO1 inhibition leads to a reduction in viral replication and could be used as a prophylactic treatment. Moreover, a protective effect of CRM1 inhibition against the cytokine storm and the associated pathological changes in lung and nasal tissues was observed, as expected [62,208]. Currently, Selinexor is being evaluated in 2 phase II clinical trial, while Verdinexor showed positive results in a Phase I trial. 

To conclude, it is interesting to note that a fine control of CRM1 in time and space could help controlling viral infection and its pathological effects: in the early stages of the infection, it is involved in the activation of the innate immune response, whereas in the next stages of the infection, it is hijacked by viruses and favors the viral replication. Finally, the modulation of CRM1 availability can dampen a further inflammatory overrun, responsible for virus associated immunopathology, at the root of the disease severity of respiratory infections. 

## Abbreviations

Kaps	Karyopherin
CRM1	Chromosome Region Maintenance 1
NPC	nuclear pore complex
SNUPN	Snuportin
LMB	Leptomycin B
NES	nuclear export signal
SINE	selective inhibitors of nuclear export
mRNP	messenger ribonucleo-particle
ARE	AU-rich element
3′UTR	3′ untranslated-region
IFN	interferon
IL	interleukine
vRNA	viral RNA
HIV-1	human immunodeficiency virus type 1
MMTV	mouse mammary tumor virus
HTLV-1	human T-cell leukemia virus type 1
NLS	nuclear localization signal
IAV	Influenza A virus
FV	foamy virus
MMPV	Mason–Pfizer Monkey Virus
HBV	Hepatitis B virus
TuMV	turnip mosaic virus
RSV	respiratory syncytial virus
SARS-CoV (-2)	severe acute respiratory syndrome coronavirus (-2)
EMCV	encephalomyocarditis virus
TMEV	Theiler’s murine encephalomyelitis virus
HCV	Hepatitis C virus
HAdV	human adenovirus
VEE	Venezuelan equine encephalitis virus
DEN	Dengue virus
CHIKV	Chikungunya virus
BDV	Borna disease virus
HPIV-2	human parainfluenza virus
IDV	Influenza D virus
CMV	cytomegalovirus
KHSV	Kaposi’s sarcoma associated herpesvirus
HSV-1	herpes simplex-virus type 1
BHV-1	bovine herpesvirus type 1
HAdV	human adenovirus
CAV	chicken anemia virus
MVM	murine minute virus
